# Alcohol Intake in Pregnancy Increases the Child's Risk of Atopic Dermatitis. The COPSAC Prospective Birth Cohort Study of a High Risk Population

**DOI:** 10.1371/journal.pone.0042710

**Published:** 2012-08-15

**Authors:** Charlotte Giwercman Carson, Liselotte Brydensholt Halkjaer, Signe Marie Jensen, Hans Bisgaard

**Affiliations:** Copenhagen Prospective Studies on Asthma in Childhood, Health Sciences, University of Copenhagen, Copenhagen University Hospital, Gentofte, Copenhagen, Denmark; UCL Institute of Child Health, University College London, United Kingdom

## Abstract

**Background:**

Atopic dermatitis has increased four-fold over the recent decades in developed countries, indicating that changes in environmental factors associated with lifestyle may play an important role in this epidemic. It has been proposed that alcohol consumption may be one contributing risk factor in this development.

**Objective:**

To analyze the impact of alcohol intake during pregnancy on the development of atopic dermatitis during the first 7 years of life.

**Method:**

The COPSAC cohort is a prospective, longitudinal, birth cohort study of 411 children born to mothers with a history of asthma, followed up for 7 years with scheduled visits every 6 months as well as visits for acute exacerbations of atopic dermatitis. Risk of atopic dermatitis from any alcohol consumption during pregnancy was analyzed as time-to-diagnosis and adjusted for known risk factors.

**Results:**

177 of 411 children developed atopic dermatitis before age 7 years. We found a significant effect of alcohol intake during pregnancy on atopic dermatitis development (HR 1.44, 95% CI 1.05–1.99 p = 0.024). This conclusion was unaffected after adjustment for smoking, mother's education and mother's atopic dermatitis.

**Limitations:**

The selection of a high-risk cohort, with all mothers suffering from asthma, and all children having a gestational age above 35 weeks with no congenital abnormality, systemic illness, or history of mechanical ventilation or lower airway infection.

**Conclusion:**

Alcohol intake by pregnant women with a history of asthma, is significantly associated with an increased risk for the child for developing atopic dermatitis during the first 7 years of life.

## Introduction

Atopic dermatitis (AD) has increased fourfold over the last five decades in western countries[1]–[4], indicating that changes in environmental factors associated with western lifestyle may play an important role[4]–[6]. It has been proposed that alcohol consumption may be one contributing factor to the rise in atopic diseases[7], [8] as alcohol consumption is part of the western lifestyle and has increased in the same period with an annual consumption nearly tripling in Denmark since 1955[9], [10]. Also, alcohol consumption during pregnancy is frequent in westernized countries[11]–[14].

We previously reported a cross-sectional analysis of a comprehensive range of potential risk factors for the development of AD by age 3 suggesting mother's AD, father's allergic rhinitis, child's FLG status, dog exposure at birth and baby's birth length as significant risk factors. Furthermore, breast feeding and mother's intake of alcohol during pregnancy was found to be marginally significant risk factors for AD in the child[15]. Recently, we reported a longitudinal analysis of age-at-onset showing that breast feeding increased the risk of AD[16].

In this 7-year follow-up study we have used close longitudinal observations to re-test our initial observation of a borderline significant risk from alcohol intake by the pregnant mother on the development of AD in the child.

## Methods

### Ethics Statement

The Copenhagen Study on Asthma in Childhood (COPSAC) was conducted in accordance with the guiding principles of the Declaration of Helsinki, approved by the Ethics Committee for Copenhagen (KF 01–289/96) and The Danish Data Protection Agency (2002–41–2434), and in compliance with “Good Clinical Practice” (GCP) guidelines. Informed, written consent was obtained from all parents.

### Participants

The COPSAC cohort is a prospective longitudinal birth cohort, including 411 children born to mothers with asthma. Mother's asthma was defined as doctor diagnosed asthma with a history of medication during two seasons[17]. The main recruiting area of the cohort was greater Copenhagen, Denmark and all children were born between August 1998 and December 2001. The children were enrolled at one month of age and visited the COPSAC clinic at scheduled visits every six months thereafter, as well as for any acute complaints from skin or airways. Skin examinations, diagnoses and treatment of AD were handled in accordance with predefined standard operating procedures by trained medical doctors employed for this purpose in the COPSAC clinic. The enrolled children were primarily cared for at the COPSAC clinic. Data validity was assured by quality control procedures. Data were collected on-line and locked after external monitoring and with an automated audit trail showing operations on the database after locking. The study was previously described in details elsewhere[17], [18]. See Table S1 for baseline characteristics.

### Risk assessments

COPSAC provides comprehensive assessments of the prenatal, perinatal and postnatal milieu, aiming to elucidate critical factors driving atopic disease expression. In this study we analyzed alcohol intake during pregnancy for development of AD during the first 7 years of life.

AD was defined based on the criteria of Hanifin and Rajka[19] as previously detailed[20], with age at first AD diagnosis used as outcome.

Childhood asthma was diagnosed based on preasthma (recurrent episodes of troublesome wheezing, breathlessness and/or cough) and the response and subsequent relapse to a 3-month trial of inhaled corticosteroids[17].

Rhinitis was defined as troublesome sneezing, blocked or runny nose in the past 12 months in periods without accompanying cold or flu[21]. All diagnoses were performed by the doctors in the COPSAC clinic.

Alcohol intake during pregnancy was determined at the interview at the 1-month visit. The mothers were interviewed about their drinking habits, and the average alcohol intake pr. week pr. trimester was entered online. Mothers drinking minimum one unit of alcohol pr. week in minimum one of the 3 trimesters were defined as having an intake of alcohol during pregnancy. One unit of alcohol was defined as 15 ml or 12 g of alcohol, explained to the mothers as e.g. one beer or one glass of wine. Information about mother's education (high school/medium-long education/university), smoking habits in the 3rd trimester (smoking yes/no) and AD (doctor diagnosed) was also recorded at the 1-month scheduled visits in the COPSAC clinic.

### Statistical analysis

The association between alcohol intake during pregnancy and development of AD during the first 7 years of life was examined by survival analysis. The children were retained in the analysis from birth, until age at first diagnosis of AD, drop-out, or 7 years of age whichever came first.

Kaplan-Meier curves were estimated for alcohol drinking during pregnancy. The plots were used as a descriptive presentation of the results, illustrating the cumulative risk of developing AD with respect to mother's alcohol intake during pregnancy. Survival analyses were performed by use of Cox proportional-hazards regression, supporting the Kaplan-Meier curves with information about hazard ratios (HR), 95% confidence intervals (CI) and p-values. The dependent variable was the time to first event (AD diagnosis, dropout or 7 years). The confounders, mother's education, smoking habits in 3rd trimester and AD, were placed in the regression models simultaneously. Tests for functional form and proportional hazards were based on martingales.

The overall significance level used was 0.05. The analyses were done using PROC TPHREG in SAS version 9.1 as well as R version 2.6.1.

## Results

COPSAC enrolled 411 infants out of which 77 were lost to follow-up before debut of AD symptoms and/or before the age of 7 years. There was no difference in alcohol consumption between the group lost to follow-up and the remaining families (Table S1). Forty eight percent of the mothers had a history of AD, 16% and 13% of the fathers had a history of asthma and AD, respectively. There were 16 siblings and 18 twins in the cohort, thus totaling 8.3% of the participants. All 411 children were included in the univariate analysis. Data for mother's education were missing for 34 children, leaving 377 children for the confounder adjusted analysis (92% of the enrolled cohort).

108 mothers in the cohort (26%) had an intake of one or more units of alcohol pr. week in minimum one of the 3 trimesters, respectively 88, 73 and 73 women in the 1^st^, 2^nd^ and 3^rd^ trimester. The quantity of alcohol intake was distributed as follows (in units of alcohol pr. trimester):

- 1^st^ trimester: range 1–7, median: 1, mean: 1.55, interquartile range: 1–2
- 2^nd^ trimester: range 1–7, median: 1, mean: 1.44, interquartile range: 1–2
- 3^rd^ trimester: range 1–7, median: 1, mean: 1.51, interquartile range: 1–2

One hundred seventy-seven children of the 411 participating infants (43%) had an AD diagnosis before age 7 years. In the group of children developing AD 31% (55) of the mothers had been drinking alcohol at some point during pregnancy, i.e. more than an average of one unit of alcohol pr. week in minimum one of the three trimesters. Of the 234 children who did not develop AD, 23% (53) of the mothers had been drinking alcohol at some point during pregnancy. This difference in prevalence of mother's alcohol drinking in the two groups of children (31% vs. 23%) was marginally statistically significant (p = 0.055)

**Figure 1:**
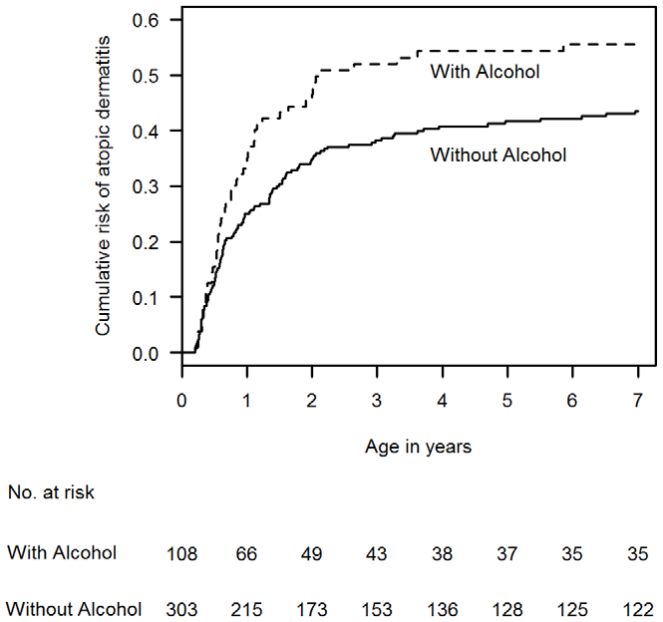
Kaplan-Meier plots for the effect of alcohol intake during pregnancy on subsequent atopic dermatitis development in the offspring during the first 7 years of life.

The age-at-onset analysis confirmed an increased risk of AD associated with alcohol intake during pregnancy, with the effect persisting throughout the whole 7 years follow-up period (Figure 1) (HR 1.44, 95% CI 1.05–1.99, p = 0.024).

The hazard ratio from the univariate analysis was largely unchanged after confounder adjusted analysis adjusting for mother's education, smoking habits 3^rd^ trimester and AD (HR 1.45, 95% CI 1.04–2.02, p = 0.029).

There was no association between alcohol intake during pregnancy and other atopic endpoints (wheeze episodes, asthma, allergic rhinitis, blood eosinophil count, total IgE, sensitization (specific IgE≥0.35 kU/L), nasal eosinophilia and cord blood IgE (IgE≥0.5 kU/L)). Results are not shown.

## Discussion

### Main finding

In this prospective, longitudinal, birth cohort study we find alcohol intake during pregnancy to be associated with an increased risk of developing AD in the first 7 years of life, adjusted for possible confounders. The increased risk was persistent throughout the whole 7 years follow-up period.

### Strengths and limitations

Recall bias is reduced from the prospective data collection, with half-yearly clinic visits and visits at any acute symptoms from the skin. The alcohol related questions were recorded before any onset of atopic diseases including AD.

Misclassification of AD is reduced by the participants being diagnosed and treated at the COPSAC clinic and not by their general practitioner. Furthermore, all mothers in the cohort have personal experience with atopic diseases, and should therefore be expected to be better at reporting symptoms. Ambiguities over the definition of AD, especially during infancy and early childhood, make the estimates of prevalence unreliable and render comparisons between studies difficult. In this cohort study the specificity of the AD diagnosis is high, since the diagnosis, detailed phenotype and management of skin lesions were controlled solely by the COPSAC clinic physicians from predefined standard operating procedures and included assessment of localization and severity at each visit. This reduces the risk of misclassification and is of particular importance in the clinical evaluation of AD where inter-observer variation is a problem[22]. The specificity of the diagnosis in this study is illustrated by the observation that the cumulative incidence of seborrheic dermatitis capitis was not significantly different in children with AD and in children with skin lesions not fulfilling AD diagnosis, suggesting that there was no misclassification between AD and seborrheic dermatitis capitis.

We have used predefined questions concerning alcohol consumption, recording average alcohol intake per week per trimester. We are aware that alcohol histories are notoriously unreliable. However, this unreliability is not biased as it must be expected to be equal among mothers to children later developing AD and not. Therefore, it can only result in a type 2 and not a type 1 error, i.e. it could have obscured an effect but it could not cause a false effect to be seen.

We have chosen to present data as a binary variable just to determine whether alcohol intake during pregnancy correlates to AD at all. We found similar estimates in sub-analyses stratifying for alcohol amount or onset/duration of alcohol intake, but the number of individuals in each group was very small. Therefore, these results are not included in the article. This narrow definition of alcohol intake limits our results and future studies designed to determine quantity of alcohol ingested should be sought. The external validity of our study is limited from the selection of a high-risk cohort, with all mothers suffering from asthma, and all children having a gestational age above 35 weeks with no congenital abnormality, systemic illness, or history of mechanical ventilation or lower airway infection. Therefore, our results need replication in an unselected population.

### Interpretation

We have previously demonstrated risk factors in the development of AD in a cross-sectional analysis by age 3 in this birth cohort[15]. We found that mother's AD, father's allergic rhinitis, child's FLG status, dog exposure at birth and higher birth length were significant factors predicting AD by 3 years of age. Duration of breastfeeding and mother's alcohol consumption during pregnancy also increased the risk of AD but were not statistical significant in this cross-sectional analysis. However, age-of-onset survival statistics was used in subsequent analyses to show, that both breast feeding[16] and now also mother's alcohol intake in pregnancy were significant risk factors for AD in the off-spring.

AD has increased dramatically since the 1960's in Western countries[1]–[3], [23] and genetic factors only cannot explain the recent rapid increase in the incidence. The exposure to early life style factors is thought to be crucial for the later development of AD, and suggestions about factors influencing the intra uterine milieu have been made[24]–[30]. A questionnaire based cohort study, reported a significant and dose-dependent effect from the mother's intake of alcohol during pregnancy in the risk of developing AD during the first 2 months of child life, but not beyond this period. This effect was mainly seen in high-risk infants (two parents with allergic disease)[31].The authors interpreted their observations to be indirectly supported by a study investigating the influence of environmental factors on levels of total IgE in cord blood of 2631 newborn infants[32]. They found an independent positive dose–response relationship between maternal alcohol consumption during pregnancy and the levels of cord blood IgE, and hypothized that the increased IgE synthesis may contribute to the immunologic sensitization. However, increased IgE is generally not believed to be directly associated to AD, but rather to illustrate a common mechanism for the atopic diseases. This is also supported by the paradox, that alcohol consumption is found associated with an increased risk of developing perennial allergic rhinitis, but not seasonal allergic rhinitis[33]. In our study we do not find any association to other atopic diseases, including cord blood IgE, allergic rhinitis, total IgE and sensitization, and can therefore not support the hypothesis that the association between alcohol and AD are caused by a higher level of IgE.

In this study we adjusted for reasonable confounders including mother's education, smoking habits in 3rd trimester and AD. These confounders were chosen, because we believe them to be the only factors both affecting the child's risk of developing AD and the mother's alcohol intake. Other factors, such as prenatal behavior, pets in home at birth and breastfeeding were not included in the analysis as confounders, because we do not believe them to affect both the exposure (mother's alcohol intake during pregnancy) and the outcome (AD). We detect an association between alcohol intake during pregnancy and the child's risk of developing AD, but the underlying causal relationship is not clear and further studies are needed to confirm our findings and explore the reasons for our observed associations.

It has been suggested that factors, which influence cytokine production by the feto-placental unit, may be important determinants of atopic disease[34]. This is further supported by findings that the effect of the maternal line on childhood AD is greater than that of the paternal line[35]–[38], which are, however, not confirmed in all studies[39], [40]. Alcohol may trigger hypersensitivity reactions, i.e. allergic, asthmatic or eczematous symptoms. The mechanisms underlying these reactions are unknown, but are hypothesized to be due to a histamine-releasing effect of acetaldehyde[41]–[46]. Gonzalez-Quintela *et al* [47] have reported that alcohol intake may induce changes in the cytokine profile, including increasing level of some Th2-associated cytokines, and impairment of the Th1 lymphocyte response. This is supported by other studies[45], [46]. However, such differences in cytokine profiles are most often found in alcohol abusers, and one should therefore be cautious in transferring these findings to our cohort's pregnant women. Still, it could be worth considering as a possible explanation for our observed association between mother's alcohol intake and the offspring's risk of developing AD, although further studies are needed to clarify this.

### Conclusion

We found that alcohol intake during pregnancy in asthmatic women is associated with a statistically significantly higher risk of developing AD in the offspring. The increased risk is still significant after confounder adjustment for other known risk factors. However, the underlying mechanism is not clear.

## Supporting Information

Table S1 **Baseline description in the full cohort and stratified according to drop-out status.**
(DOC)

## References

[1] Schultz LF, Hanifin JM (1992) Secular change in the occurrence of atopic dermatitis. Acta Derm Venereol Suppl (Stockh) 176: 7–12. 1476042

[2] Schultz LF, Diepgen T, Svensson A (1996) The occurrence of atopic dermatitis in north Europe: an international questionnaire study. J Am Acad Dermatol 34: 760–764. 8632070 10.1016/s0190-9622(96)90009-2

[3] Olesen AB, Ellingsen AR, Olesen H, Juul S, Thestrup-Pedersen K (1997) Atopic dermatitis and birth factors: historical follow up by record linkage. BMJ 314: 1003–1008. 9112844 10.1136/bmj.314.7086.1003 PMC2126413

[4] Law M, Morris JK, Wald N, Luczynska C, Burney P (2005) Changes in atopy over a quarter of a century, based on cross sectional data at three time periods. BMJ 330: 1187–1188. 15833748 10.1136/bmj.38435.582975.AE PMC558016

[5] Krause T, Koch A, Friborg J, Poulsen LK, Kristensen B, et al (2002) Frequency of atopy in the Arctic in 1987 and 1998. Lancet 360: 691–692. 12241878 10.1016/s0140-6736(02)09841-0

[6] Von Hertzen LC, Haahtela T (2004) Asthma and atopy - the price of affluence? Allergy 59: 124–137. 14763924 10.1046/j.1398-9995.2003.00433.x

[7] Vally H, Thompson PJ (2003) Alcoholic drink consumption: a role in the development of allergic disease? Clin Exp Allergy 33: 156–158. 12580904 10.1046/j.1365-2222.2003.01604.x

[8] Linneberg A, Petersen J, Nielsen NH, Madsen F, Frolund L, et al (2003) The relationship of alcohol consumption to total immunoglobulin E and the development of immunoglobulin E sensitization: the Copenhagen Allergy Study. Clin Exp Allergy 33: 192–198. 12580911 10.1046/j.1365-2222.2003.01515.x

[9] Fagt S, Trolle E (2001) [The supply of food in 1955–1999. Development in Danish Diet - consumption, food, purchases and eating habits] (danish). 38–40.

[10] Academy of Medical Sciences (2004) Calling Time - The Nation's drinking as a major health issue.

[11] Olsen J, Frische G, Poulsen AO, Kirchheiner H (1989) Changing smoking, drinking, and eating behaviour among pregnant women in Denmark. Evaluation of a health campaign in a local region. Scand J Soc Med 17: 277–280. 2602919 10.1177/140349488901700404

[12] Ogston SA, Parry GJ (1992) EUROMAC. A European concerted action: maternal alcohol consumption and its relation to the outcome of pregnancy and child development at 18 months. Results–strategy of analysis and analysis of pregnancy outcome. Int J Epidemiol 21 Suppl 1S45–S71. 1399220 10.1093/ije/21.supplement_1.s45

[13] Kesmodel U, Wisborg K, Olsen SF, Henriksen TB, Secher NJ (2002) Moderate alcohol intake during pregnancy and the risk of stillbirth and death in the first year of life. Am J Epidemiol 155: 305–312. 11836194 10.1093/aje/155.4.305

[14] Ebrahim SH, Luman ET, Floyd RL, Murphy CC, Bennett EM, et al (1998) Alcohol consumption by pregnant women in the United States during 1988–1995. Obstet Gynecol 92: 187–192. 9699749 10.1016/s0029-7844(98)00205-1

[15] Bisgaard H, Halkjaer LB, Hinge R, Giwercman C, Palmer C, et al (2009) Risk analysis of early childhood eczema. J Allergy Clin Immunol 123: 1355–1360. 19501236 10.1016/j.jaci.2009.03.046

[16] Giwercman C, Halkjaer LB, Jensen SM, Bonnelykke K, Lauritzen L, et al (2010) Increased risk of eczema but reduced risk of early wheezy disorder from exclusive breast-feeding in high-risk infants. J Allergy Clin Immunol 125: 866–871. 20236698 10.1016/j.jaci.2010.01.026

[17] Bisgaard H (2004) The Copenhagen Prospective Study on Asthma in Childhood (COPSAC): design, rationale, and baseline data from a longitudinal birth cohort study. Ann Allergy Asthma Immunol 93: 381–389. 15521375 10.1016/S1081-1206(10)61398-1

[18] Bisgaard H, Hermansen MN, Loland L, Halkjaer LB, Buchvald F (2006) Intermittent inhaled corticosteroids in infants with episodic wheezing. N Engl J Med 354: 1998–2005. 16687712 10.1056/NEJMoa054692

[19] Hanifin JM, Rajka G (1980) Diagnostic features of atopic dermatitis. Acta Derm Venereol 92: 44–47.

[20] Halkjaer LB, Loland L, Buchvald FF, Agner T, Skov L, et al (2006) Development of atopic dermatitis during the first 3 years of life: the Copenhagen prospective study on asthma in childhood cohort study in high-risk children. Arch Dermatol 142: 561–566. 16702493 10.1001/archderm.142.5.561

[21] Chawes BL, Kreiner-Moller E, Bisgaard H (2009) Objective assessments of allergic and nonallergic rhinitis in young children. Allergy 64: 1547–1553. 19663868 10.1111/j.1398-9995.2009.02085.x

[22] Williams HC, Burney PG, Strachan D, Hay RJ (1994) The U.K. Working Party's Diagnostic Criteria for Atopic Dermatitis. II. Observer variation of clinical diagnosis and signs of atopic dermatitis. Br J Dermatol 131: 397–405. 7918016 10.1111/j.1365-2133.1994.tb08531.x

[23] Law M, Morris JK, Wald N, Luczynska C, Burney P (2005) Changes in atopy over a quarter of a century, based on cross sectional data at three time periods. BMJ 330: 1187–1188. 15833748 10.1136/bmj.38435.582975.AE PMC558016

[24] Jones CA, Holloway JA, Warner JO (2000) Does atopic disease start in foetal life? Allergy 55: 2–10. 10696851 10.1034/j.1398-9995.2000.00109.x

[25] Bjorksten B (1999) The intrauterine and postnatal environments. J Allergy Clin Immunol 104: 1119–1127. 10588990 10.1016/s0091-6749(99)70002-3

[26] Bjorksten B (1999) Environment and infant immunity. Proc Nutr Soc 58: 729–732. 10604209 10.1017/s0029665199000956

[27] Xu B, Jarvelin MR, Pekkanen J (1999) Prenatal factors and occurrence of rhinitis and eczema among offspring. Allergy 54: 829–836. 10485386 10.1034/j.1398-9995.1999.00117.x

[28] Kurzius-Spencer M, Halonen M, Carla L, I, Martinez FD, Wright AL (2005) Prenatal factors associated with the development of eczema in the first year of life. Pediatr Allergy Immunol 16: 19–26. 15693907 10.1111/j.1399-3038.2005.00233.x

[29] Prescott SL (2003) Early origins of allergic disease: a review of processes and influences during early immune development. Curr Opin Allergy Clin Immunol 3: 125–132. 12750609 10.1097/00130832-200304000-00006

[30] Bjorksten B (1999) Allergy priming early in life. Lancet 353: 167–168. 9923869 10.1016/S0140-6736(05)77212-3

[31] Linneberg A, Petersen J, Gronbaek M, Benn CS (2004) Alcohol during pregnancy and atopic dermatitis in the offspring. Clin Exp Allergy 34: 1678–1683. 15544590 10.1111/j.1365-2222.2004.02101.x

[32] Bjerke T, Hedegaard M, Henriksen TB, Nielsen BW, Schiotz PO (1994) Several genetic and environmental factors influence cord blood IgE concentration. Pediatr Allergy Immunol 5: 88–94. 8087193 10.1111/j.1399-3038.1994.tb00223.x

[33] Bendtsen P, Gronbaek M, Kjaer SK, Munk C, Linneberg A, et al (2008) Alcohol consumption and the risk of self-reported perennial and seasonal allergic rhinitis in young adult women in a population-based cohort study. Clin Exp Allergy 38: 1179–1185. 18294256 10.1111/j.1365-2222.2008.02945.x

[34] Macaubas C, de Klerk NH, Holt BJ, Wee C, Kendall G, et al (2003) Association between antenatal cytokine production and the development of atopy and asthma at age 6 years. Lancet 362: 1192–1197. 14568741 10.1016/s0140-6736(03)14542-4

[35] Bradley M, Kockum I, Soderhall C, Van Hage-Hamsten M, Luthman H, et al (2000) Characterization by phenotype of families with atopic dermatitis. Acta Derm Venereol 80: 106–110. 10877129

[36] Ruiz RG, Kemeny DM, Price JF (1992) Higher risk of infantile atopic dermatitis from maternal atopy than from paternal atopy. Clin Exp Allergy 22: 762–766. 1525695 10.1111/j.1365-2222.1992.tb02816.x

[37] Moore MM, Rifas-Shiman SL, Rich-Edwards JW, Kleinman KP, Camargo CA Jr, et al (2004) Perinatal predictors of atopic dermatitis occurring in the first six months of life. Pediatrics 113: 468–474. 14993536 10.1542/peds.113.3.468 PMC1488729

[38] Harris JM, Cullinan P, Williams HC, Mills P, Moffat S, et al (2001) Environmental associations with eczema in early life. Br J Dermatol 144: 795–802. 11298539 10.1046/j.1365-2133.2001.04135.x

[39] Bohme M, Wickman M, Lennart NS, Svartengren M, Wahlgren CF (2003) Family history and risk of atopic dermatitis in children up to 4 years. Clin Exp Allergy 33: 1226–1231. 12956743 10.1046/j.1365-2222.2003.01749.x

[40] Wadonda-Kabondo N, Sterne JA, Golding J, Kennedy CT, Archer CB, et al (2004) Association of parental eczema, hayfever, and asthma with atopic dermatitis in infancy: birth cohort study. Arch Dis Child 89: 917–921. 15383434 10.1136/adc.2003.034033 PMC1719677

[41] Vally H, Thompson PJ (2003) Allergic and asthmatic reactions to alcoholic drinks. Addict Biol 8: 3–11. 12745410 10.1080/1355621031000069828

[42] Linneberg A, Berg ND, Gonzalez-Quintela A, Vidal C, Elberling J (2008) Prevalence of self-reported hypersensitivity symptoms following intake of alcoholic drinks. Clin Exp Allergy 38: 145–151. 17927799 10.1111/j.1365-2222.2007.02837.x

[43] Vally H, de KN, Thompson PJ (2000) Alcoholic drinks: important triggers for asthma. J Allergy Clin Immunol 105: 462–467. 10719294 10.1067/mai.2000.104548

[44] Nihlen U, Greiff LJ, Nyberg P, Persson CG, Andersson M (2005) Alcohol-induced upper airway symptoms: prevalence and co-morbidity. Respir Med 99: 762–769. 15878494 10.1016/j.rmed.2004.11.010

[45] Cook RT (1998) Alcohol abuse, alcoholism, and damage to the immune system–a review. Alcohol Clin Exp Res 22: 1927–1942. 9884135

[46] Kazbariene B, Kalibatas J, Krikstaponiene A, Zabulyte D, Monceviciute-Eringiene E (2007) Alterations of human immune system functions in relation to environmental contamination, gender and alcohol consumption intensity. Cent Eur J Public Health 15: 13–17. 17491553 10.21101/cejph.a3404

[47] Gonzalez-Quintela A, Vidal C, Lojo S, Perez LF, Otero-Anton E, et al (1999) Serum cytokines and increased total serum IgE in alcoholics. Ann Allergy Asthma Immunol 83: 61–67. 10437818 10.1016/S1081-1206(10)63514-4

