# Supplementary material for: Alcohol Intake in Pregnancy Increases the Child's Risk of Atopic Dermatitis. The COPSAC Prospective Birth Cohort Study of a High Risk Population
Source: PLoS One. 2012 Aug 15;7(8):e42710. doi: 10.1371/journal.pone.0042710 (PMC3419732; doi:10.1371/journal.pone.0042710)
Supplement: Table S1 — Baseline description in the full cohort and stratified according to drop-out status. (DOC) [file pone.0042710.s001.doc]

**Supporting information table S1: Baseline description in the full cohort and stratified according to drop-out status**

|  | **Full cohort** | **Follow up 0-7 years** | **Lost to follow-up** | **P-value** | |
| --- | --- | --- | --- | --- | --- |
|  |  |  |  |  |  |
| **Numbers, N:** | 411 | 334 | 77 | - |  |
|  |  |  |  |  |  |
| **Drop-out reasons, N:**      Death  Disabling disease      Social (removed from parents)      Emotional distress to study procedures      Emigration      Parental lack of time      No specified reason |  |  | 2  2  1  4  7  3  58 |  |  |
|  |  |  |  |  |  |
| **Genetics:** |  |  |  |  |  |
| Male, % | 49 | 50 | 45 | NS | c |
| Caucasian, % | 97 | 97 | 95 | NS | c |
| FLG null mutation^1^, % | 11 | 11 | 8 | NS | c |
|  |  |  |  |  |  |
| **Atopic heredity:** |  |  |  |  |  |
| Mother asthma, % | 100 | 100 | 100 | - |  |
| Mother allergic rhinitis, % | 76 | 78 | 67 | NS | c |
| Mother eczema, % | 48 | 51 | 36 | **0.02** | c |
| Father asthma, % | 17 | 20 | 4 | **0.001** | c |
| Father allergic rhinitis, % | 33 | 37 | 13 | **<0.001** | c |
| Father eczema, % | 13 | 14 | 11 | NS | c |
|  |  |  |  |  |  |
| **Socio-demographics:** |  |  |  |  |  |
| Household income, mean (SD),      1000 DKK | 494 (207) | 505 (205) | 425 (207) | **0.009** | t |
| Mother’s education      Low, %      Inter-medium, %      High, % | 60  27  14 | 59  27  13 | 61  20  18 | NS | c |
| Mother’s occupation      Student, %      Unemployed, %      Non-professional, %      Professional, % | 11  10  34  46 | 11  9  33  47 | 13  17  35  35 | NS | c |
| Urban living, % | 93 | 94 | 91 | NS | c |
| Father’s age, mean (SD), yr | 32 (5.2) | 32.3 (5.0) | 30.7 (5.7) | **0.03** | t |
| Mother’s age, mean (SD), yr | 30 (4.5) | 30.3 (4.4) | 28.9 (5.0) | **0.03** | t |
|  |  |  |  |  |  |
| **Birth:** |  |  |  |  |  |
| Birth weight, mean (SD), Z-score | 0.06 (1.12) | 0.10 (1.12) | -0.13 (1.10) | NS | t |
| Birth length, mean (SD), Z-score | 0.12 (1.14) | 0.14 (1.14) | 0.01 (1.18) | NS | t |
| Birth BMI, mean (SD), Z-score | 0.00 (1.03) | 0.04 (1.03) | -0.19 (1.01) | NS | t |
| Gestational age, mean (SD), wks | 39.9 (1.6) | 39.9 (1.6) | 39.9 (1.4) | NS | t |
| Cesarean sectio, % | 21 | 21 | 18 | NS | c |
| Apgar score >9, % | 97 | 96 | 99 | NS | c |
| Season of birth      Winter, %      Spring, %      Summer, %      Fall, % | 23  21  27  29 | 23  20  28  30 | 23  26  25  26 | NS | c |
|  |  |  |  |  |  |
| **Prenatal exposures:** |  |  |  |  |  |
| Alcohol in pregnancy, % | 26 | 27 | 24 | NS | c |
| Mother smoking in 3^rd^ trimester, % | 15 | 13 | 25 | **0.01** | c |
| Antibiotic use in 3^rd^ trimester, % | 14 | 14 | 14 | NS | c |
| Paracetamol use in 3^rd^ trimester, % | 14 | 16 | 8 | NS | c |
|  |  |  |  |  |  |
| **Postnatal exposures:** |  |  |  |  |  |
| Solely breastfed > 4wks, % | 82 | 83 | 76 | NS | c |
| Duration of solely breastfeeding,  mean (SD), days | 113 (62) | 115 (62) | 96 (61) | NS | t |
| Daycare, age at start, median (IQR) | 346 (246-433) | 344 (245-429) | 368 (260-483) | NS | w |
| Cat in household 1^st^ yr, % | 14 | 14 | 10 | NS | c |
| Dog in household 1^st^ yr, % | 13 | 14 | 6 | NS | c |
| Older siblings      0, %      1, %      2+, % | 61  28  11 | 59  29  12 | 70  20  9 | NS | c |
| Nicotine in hair at 1 yr, median (IQR) | 0.7 (0.3-2.5) | 0.7 (0.3-2.2) | 1.3 (0.5-6.1) | **0.005** | w |
|  |  |  |  |  |  |
|  |  |  |  |  |  |
| 1: FLG genotyping was performed for R501X and 2282del4  c: chi-square test | | | | | |
| t: unpaired T-test |  |  |  |  |  |
| w: Wilcoxon rank sum test |  |  |  |  |  |
| IQR: Inter-Quartile Range |  |  |  |  |  |
| NS*: P*>0.05 |  |  |  |  |  |
